# Case of Epilepsy

**Published:** 1830

**Authors:** J. M. Staughton

**Affiliations:** Late Professor of Surgery in the Medical Department of the Columbian College


ART—IV. *Case of Epilepsy, communicated by* J. M. STAUGHTON, M. D. *Late Professor of Surgery in the Medical Department of the Columbian College.*

In the summer of 1827, a gentleman of about fifty-three years of age and of sedentary habits, as he was walking from Washington City to Georgetown, suddenly fell to the ground and was violently convulsed. After a few minutes he came to himself, and walked, not without much difficulty, to his lodgings in Georgetown, a distance of about half a mile. He was put to bed and shortly after had another convulsion, which was followed by great exhaustion, during which the contents of the bladder and rectum were involuntarily discharged. I was sent for, and saw him that evening. I found him very weak from his convulsions, his pulse was full and soft, and there was nothing to indicate the slightest apoplectic tendency. His mind was unimpaired and though he confessed himself much alarmed, coolly reasoned on the consequences. He was a man of great regularity in his mode of life, and his tongue did not seem to indicate that the epilepsy was of a gastric origin ; after directing a stimulating pediluvium, I left him. The next morning he was better, sitting up and trying to write. He found that he could not command the motions of the right hand, and shewed me with great concern the almost illegible lines that he had written. This gradually vanished and in a few days he was able to walk over to my house, a distance of two miles, thinking himself perfectly recovered. A week or ten days after this, as he was walking along Pennsylvania Avenue, another convulsion prostrated him. He was carried into an adjoining house and some one bled him. The venesection appeared to cause a repetition of the convulsion. He was put to bed and a man was hired to attend him. When I saw him the next morning he addressed me by name, then suddenly turned on his left side and the whole right side of his body underwent a powerful convulsion which lasted about a minute and then gradually abated. From the consequent debility he slowly aroused, and entered into free conversation, which was arrested in about ten minutes by a similar convulsion, lasting much about the same length of time as the preceding, and which left him much in the same state. His attendant told me that these convulsions had succeeded each other during the whole night at intervals of ten or fifteen minutes and that they resembled those that I had first witnessed. The patient's pulse was weak, and small, and quick. The skin, tongue, and countenance were natural. The bowels were constipated; during the convulsions, the face was frightfully distorted on the right side and the eyes wildly rolled in various directions, the lids remaining wide open. There was a regular motion of the right arm at the commencement of each convulsion by which the thumb nail was brought over the forehead, ploughing up the skin and keeping the face bloody. I ought to mention more particularly that the convulsive action was confined to the right side of the body, the left remaining perfectly in a quiescent state, with the muscles flaccid, resembling in a very striking manner the effect of the *strychnos nux vomica* in Hemiplegia.

Without giving minutely the daily history of the case, I will simply state that these convulsions continued at the same intervals for nine days; his bodily vigor sinking under their exhausting effects, but his mind continuing calm and collected in the intermissions. He talked unreservedly with me on his temporal affairs, and was perfectly sensible of his dangerous state. He requested me to write to his sister in Corsica, of which island he was a native, to inform her of his disolution when it should occur. His vital energies were evidently giving way and I looked forward to his certain departure.

He was a man of highly accomplished education, a graduate of one of the most distinguished Universities of Italy, and spoke the Latin, Italian, and French with great fluency. It is very singular that from the moment of his attack he utterly forgot what little of English he had learned during his residence in Great Britain and America, nor did he ever recover it. Yet he retained a facile remembrance of the languages he had learned in his boyhood.

Notwithstanding I made use of all the therapeutic means which are universally employed in similar cases, and though I had the benefit of the advice of Drs. Mechlin and Borrows, of Washington, I am free to confess my belief that our remedies were of very little service, and that his recovery is to be ascribed to the powerful efforts of his system.

For he did recover, and the only vestige of his disease that remained was slight hemiplegia on the right side, which did not materially interfere with his walking. This, too, gradually wore off, and when, some months after, he left the city, a very slight halt in his gait indicated the relics of his paralysis.

A short time before he left Washington he communicated to me confidentially the cause of his disease, with a request that after his death I would publish his case. He is since dead in a distant land, and I now comply with his request in presenting his history to the Profession. From what I can learn he fell a victim to the baleful cause which I proceed to mention. Ecclesiasticus fuit, et regulis ecclesiæ suæ a matrimonio prohibitus. Per complurimos annos assuefactus fuit libidinis suæ de masturbatione solatium prœbere.

I am aware that this is no new case. The writings of Tissot Broussais, Fournier, and others, present many interesting cases of this character.

This miserable victim of a habit, unfortunately but too common, ascribed the failure of his memory and of his health to this cause, and bitterly did he lament that the institutions of his sect prescribed a life of celibacy which so illy corresponded with his passions and social feelings.

